# The Use of Nanomedicine for Targeted Therapy against Bacterial Infections

**DOI:** 10.3390/antibiotics8040260

**Published:** 2019-12-11

**Authors:** Abdulkader Masri, Ayaz Anwar, Naveed Ahmed Khan, Ruqaiyyah Siddiqui

**Affiliations:** 1 Department of Biological Sciences, School of Science and Technology, Sunway University, Selangor 47500, Malaysia; abdulka.m@imail.sunway.edu.my (A.M.);; 2 Department of Biology, Chemistry and Environmental Sciences, College of Arts and Sciences, American University of Sharjah, University City, Sharjah 26666, UAE

**Keywords:** nanomedicine, antibacterial efficacy, drug delivery

## Abstract

The emergence of drug resistance combined with limited success in the discovery of newer and effective antimicrobial chemotherapeutics poses a significant challenge to human and animal health. Nanoparticles may be an approach for effective drug development and delivery against infections caused by multi-drug resistant bacteria. Here we discuss nanoparticles therapeutics and nano-drug delivery against bacterial infections. The therapeutic efficacy of numerous kinds of nanoparticles including nanoantibiotics conjugates, small molecules capped nanoparticles, polymers stabilized nanoparticles, and biomolecules functionalized nanoparticles has been discussed. Moreover, nanoparticles-based drug delivery systems against bacterial infections have been described. Furthermore, the fundamental limitation of biocompatibility and biosafety of nanoparticles is also conferred. Finally, we propose potential future strategies of nanomaterials as antibacterials.

## 1. Introduction

Infectious ailments, especially due to bacteria are a significant burden on public health, killing more than 14 million people annually [1,2]. Molecular components of bacterial pathogens are extensively dissimilar from human cells such as their genetic material, ribosomes, cell membranes, cell wall and biosynthetic routes, hence these aspects are usually exploited to derive antimicrobial agents [3]. In recent years, the development of antimicrobial resistance is proving a major setback in our ability to counter morbidity and mortality associated with bacterial infections [2]. Several strategies have been employed by bacteria to resist antimicrobials including efflux pumps, enzymatic suppression by either hydrolytic degradation or chemical alterations such as the addition of a phosphate group acetylation or hydrolysis, changing target and reprogramming biological synthesis, and accelerated evolution of acquired resistance in microorganisms [4]. The danger of antibacterial resistance is a significant threat to human health worldwide [5,6]. In addition, many antibiotics like fluoroquinolone and aminoglycosides exhibit serious side effects [7]. The majority of multidrug resistance (MDR) infections need extended antibiotic therapy that are accompanied with significant health-care costs [8,9]. Hence, there is a need for the development of new effective antibacterial strategies.

Recently, the applications of nanotechnology especially the use of nanoparticles has been highlighted as a promising solution to the challenges posed by existing antimicrobials. Several nanoparticles have been utilized as delivery vehicles comprising of dendrimers, liposomes, metallic nanoparticles, and polymeric nanoparticles [10]. Size reduction methods and technologies yield different types of nanostructures that exhibit unique physicochemical properties such as magnetism, physical strength, electrical conductance, chemical reactivity and optical effects [11]. These criteria help in increasing the surface area, enhancing release of drug, reducing the dose required, and improving solubility and bioavailability of the compounds [12]. Moreover, these properties are also important for the diagnosis of microbial diseases with high sensitivity and selectivity and those with fluorescent properties or labeled with fluorescent dyes have also been employed for bacterial detection and susceptibility. In addition, inorganic nanoparticle-based diagnostic approaches depend upon the identification of known bacterial genome sequences by directing probes, and thus may not recognize altered and/or novel bacterial strains [13]. Furthermore, nanoparticles based specific drug targeting improve the therapeutic index, increase stability of drug, and decrease drug resistance. For example, silver nanoparticles affect *Escherichia coli* by forming complex with electron donor groups on amino acids and nucleic acids [12]. At present, liposomal drugs and polymer–drug conjugates have been approved for infectious diseases treatment, and many other antimicrobial nanoparticle formulations are under preclinical test [14].

Gold nanoparticles have gained significant attention recently due to their tunable antimicrobial applications. Gold has been the subject of interest against bacterial infections for its biocompatibility and ease in conjugation with drugs and biomolecules. Gold conjugated with various drugs have shown to enhance their efficacy against bacteria. Gold nanoparticles coated with aminoglycosides have been found to be efficient antibacterial agents against various bacteria such as *Staphylococcus aureus*, *Micrococcus luteus*, *E. coli* and *Pseudomonas aeruginosa* [15]. In another study, after assessment of the surface chemistry of nanoparticles, the synergistic mechanism showed that hydrophobic cationic conjugated gold nanoparticles reduced the minimum inhibitory concentration (MIC) of fluoroquinolone against multidrug resistant by 8–16 times [16]. More recently, formulations of carbapenem loaded gold nanoparticles with different sizes (35 nm, 70 nm and 200 nm) showed potent antibacterial activity against MDR bacteria including *Klebsiella pneumoniae*, *Proteus mirabilis* and *Acinetobacter baumanii*. Imipenem coated gold nanoparticles showed four-fold decrease in the MIC whereas meropenem decreased the MIC by three-fold [17]. In another report, the antibiofilm activity of gold nanoparticles of around 50 nm and nanoclusters around 2–3 nm stabilized by same ligand, 3-(diphenylphosphino) propionic acid against *Staphylococcus aureus and Streptococcus mutans* have shown that smaller nanoclusters exhibit better antibacterial effects against gram-positive bacteria [18]. The antibacterial activity of gold nanoparticles depends mainly on their cargo; however, size and shape of gold nanoparticles are also known alter the antibacterial potency. Gold nanoparticles of different shapes (sphere, rod, star and flower) can be prepared by utilizing different chemical protocols, while certain stabilizing and reducing agents can give rise to size selectivity to a very narrow range [19].

Here, we focus on several aspects of nanoparticles for enhancing treatment efficacy of compounds. The therapeutic potential of nanoparticles, drug delivery, and nanoparticles cytotoxicity will be discussed. Moreover, we discuss forthcoming promising platforms utilizing nanoparticles against bacterial infections, as well as their future prospects.

## 2. Therapeutic Efficacy of Nanoparticles

Several aspects can play a role in the therapeutic efficacy of nanoparticles, and this has led to the development of more complex nanoparticles. Many factors play a role in the reaction of nanomaterial with bacteria including hydrophobicity, static electricity attraction, van der Waals forces and receptor–ligand connection which affects the therapeutic potency [20].

In effective targeting, electrostatic interactions occurring between the negative charge of the bacteria surface and the cationic charge of nanoparticles can increase the therapeutic efficacy of nanoparticles [21]. For example, Gold nanorods or nanospheres showed electrostatic interaction with the negative charge of teichoic acid on *Bacillus cereus* [22]. Mannose substituted gold metal nanoparticles have been shown to bind with the lectin pili as a target on the surface of *E. coli* [23]. Nanomaterials can interact with intracellular components like respiratory enzymes and DNA to disrupt cellular mechanisms and electrolyte balance, resulting in bacterial lysis [24]. Moreover, the surface chemical composition of nanoparticles is crucial to modify their contact with the bacterial cellular system, improving their therapeutic index while concurrently dropping their toxicity against host cells [11]. For example, Bayraktar (2007) reported that aspartate amino acid functionalized gold nanoparticles bind to large surface of cytochrome c whereas phenylalanine conjugation exhibited much smaller binding surface on cytochrome [25]. In the passive bacterial targeting, the high vascular permeability and impaired function of lymphatic system are resulted in bacterial infection site, which lead to nanoparticles accumulations [26]. For instance, Polyethylene glycerol liposomes favorably located in an intramuscular *S. aureus* infection site [27].

### 2.1. Antibiotics Capped Nanoparticles

Antimicrobials capped nanoparticles have shown improvements in therapeutic index and pharmacokinetics of the drug compared with the free drug equivalents. These conjugates have been recently synthesized and have shown enhanced efficacy of antibiotic through the synergistic action by raising the concentration of drugs at the target site [28]. Moreover, these systems can enhance stability, bioavailability, targetability, and biological distribution to decrease the toxicity [29]. There are many ways for drug loading into nanoparticles such as chemical conjugation or physical encapsulation, adsorption [14]. For instance, in citrate reduction method, the resultant AuNPs (14 nm) is functionalized by different types of antibacterials (streptomycin, ampicillin and kanamycin). These nanoconjugates have been tested against (*E. coli*, *S. aureus* and *M. luteus*). The findings demonstrated high efficiency of antibiotics capped AuNPs compared with free antibiotics against these bacteria [30]. In another study for gold NPs conjugated with second generation cephalosporine (cefaclor), the researchers illustrated high efficacy of AuNPs-cefaclor against *E. coli* and *S. aureus* due to capping of AuNPs by amine group of cefaclor, leaving β-lactam ring unmodified for activity [31]. Furthermore, the functionalization of anionic mesoporous silica nanoparticles with positively charged Polymixn B was achieved through net result of attractive forces between amine groups of antibiotics and anions on the surface of nanoparticles. These nanoparticles resulted in improved antibacterial activity compared with unbounded Polymixn B against various Gram-negative bacteria. Moreover, cytotoxicity was reduced by reducing reactive oxygen species generation [8]. Lately by using biogenic synthesis method, Sangaonkar and Pawar (2018) conjugated small sized Ag- nanoparticles (5–30 nm) with tetracycline, and this compound displayed biocidal action against four of the seven bacteria tested [32]. The mechanism of action involved deregulation of main efflux pump (tolC) and downregulation of proteins responsible for assembling of (tolC) in *E. coli* outer membrane [33].

The stable ciprofloxacin conjugated ZnO nanoparticles showed concentration-dependent antibacterial efficacy against *E. coli* and *S. aureus*. This activity may be attributed to ZnO nanoparticles through contribution in both the interfering with function of NorA protein in *S. aureus* as pumping activity and enhancing the intake of antibiotics into *S. aureus* [34]. Whereas, the bactericidal ability of nitrofurantoin, penicillin G, and amoxicillin in combination with ZnO nanoparticles against *S. aureus* was reduced because of the possibility of forming weak hydrogen bonds with hydroxylated nanoparticles [34]. Another study of Ciprofloxacin loaded to the composite of cobalt ferrite, graphene oxide, and silver nanoparticles showed antibacterial synergistic action against both types of bacteria [35]. The MIC of the composite against *E. coli* was 2.5 mg/mL, while upon conjugation with ciprofloxacin, the MIC was lowered to 1.25 mg/mL and the composite have no important influence on cytoplasmic membrane [35]. Furthermore, Grace & Pandian (2007) showed that chelating amine groups of aminoglycoside drugs with small size of Au-nanoparticles (15 nm) illustrated high zone of inhibition compared with pure drugs against bacteria [15]. Recent study showed that cephradine conjugation with Ag nanoparticles offer a change in sensitivity of the drug and boosted the antibacterial effects against Gram-negative bacteria [36].

### 2.2. Small Molecules Conjugated Nanoparticles

The small molecules conjugated nanoparticles can facilitate development of a wide range of nanomaterials for biomedical applications. The appropriate combination between nanoparticles and small molecule chemistry can simplify growth of nanomaterials for biomedical uses, small molecule alterations could enhance binding attraction and modify biological characteristics of nanomaterials and thereby allow small molecule-mediated multivalent binding to receptors of the cell [37]. These conjugates provoke bacterial resistance more heavily compared with usual antibacterials because numerous mechanisms of nanoparticles may not be simply controlled by approaches used by microbes to grow resistance instantaneously and appear harmless to human cells [38]. For example, mixtures of distinct synthetic thiol ligands are fused with p-mercaptobenzoic acid adapted Au- nanoparticles, and this conjugate showed antibacterial effects against MDR bacteria. Also, this conjugate revealed delayed development of resistance compared to the antibiotic alone by affecting the transcription process of a number of genes [39]. Furthermore, Zhao et al. (2010) showed that amino-substituted pyrimidines presented on Au nanoparticles had antibacterial activities against MDR bacteria via chelating of Mg^2+^ or Ca^2+^ and compromising ATPase activities [38]. In another study, Anwar et al. (2018) reported enhancement of antibacterial effects of gold nanoparticles combined with 4-Dimethyl aminopyridinium propylthioacetate against *E. coli* in comparison with unmixed compound [40].

### 2.3. Polymer Stabilized Nanomaterials

Polymer stabilized nanoparticles have notable effects against bacterial infections. The dominant synthetic microbial agents until now are polymers having positively charged groups such as quaternary phosphonium or ammonium, which trigger fast adsorption into the anionic surfaces of bacteria [41,42]. Covering nanoparticles with artificial polymers have been revealed to notably decrease the attachment of plasma proteins, contact with opsonins, and clearance by the reticuloendothelial system [43]. However, several of these antimicrobials, though active against selected bacteria, are poisonous for mammalian cells [3,44]. The polymers have been used in nanoparticles preparation to increase the appropriate host response of cationic antimicrobial agents through a balanced pattern [45].

Some of the most common natural polymers used in nanomedicine are chitosan, gum acacia, xanthan gum, etc. For example, the stabilization of Ag- nanoparticles by gum acacia and loaded with hesperidin exhibited significant bactericidal effects against MRSA and *E. coli* K1 and reduced bacterial-mediated host cells cytotoxicity [46]. For different metal, Regiel et al. (2015) used the biocompatible and decomposable carbohydrate chitosan as stabilizer and reducing agent for gold nanoparticles synthesis and the resulting nanocomposite with moderate molecular mass [47]. The ultimate degree of deacetylation displayed maximum activity against bacterial biofilm establishing species of *P. aeruginosa* and *S. aureus* by producing a distracting consequence on the bacterial cell wall while their endocytosis was retarded on the eukaryotic cells [47]. Moreover, natural gallic acid conjugated with magnetite iron oxide at concentration of 100 mg/mL showed powerful bactericidal effect when tested on diverse bacterial strains. The high antibacterial potency was attributed to increased penetration of cells with impairment of cell wall by destroying β-1,4-glyosidic bond leading to cell wall decomposition [48].

On the other hand, the commonly used synthetic polymers are polyvinyl pyrimidine, polylactic acid etc. Recently, the MIC of silver nanoclusters functionalized with branched polyethylenimine (bPEI−Ag NCs) was defined against twelve pathogenic MDR bacteria [49]. The results revealed that a 10 to 15-fold lower MIC was found for nanoparticles than that of PEI and from two to three times lower than that of lonely silver nitrate and the noticed wide range antibacterial activity can be assigned to the positive character of hydrophobic surface ligands that simplify the action of bPEI−Ag NCs with bacterial cells that initiates membrane injury, besides liberation of silver ions from the nanocluster compound [49]. By using a new seed growth synthesis, the coating of bulk glass materials with silver nanoplates was achieved. This glass material coated silver nanoplates can exert an antibacterial action based on the photo-thermal effect through laser-induced hyperthermia, which can be switched on by 808 nm laser excitation in 20 min. The laser-induced hyperthermia almost completely eliminated bacteria *S. aureus* and *E. coli* [50]. Moreover, bulk glass surfaces coated with polymer with synthesized citrate silver nanoplates have ability to strongly absorb laser radiation in the near infrared (NIR) range and this coating exerts an antibacterial activity against *S. aureus* and *E. coli* [51]. The mechanism of action of these composites are still under debate but they rely on release of silver ions from nano-objects and the subsequent interaction of Ag with bacteria and the antibacterial action caused by photothermal effect [50,51].

### 2.4. Biomolecules Functionalized Nanoparticles

Biomolecules show particular and potent corresponding interactions that can communicate biological specificity to nanoparticles. Nanoparticles can be easily conjugated with biomolecules including peptides, proteins, nucleotides, vaccines and drugs. The traditional antimicrobial peptides (AMPs) derived from endogenous peptides suffer essential obstacles in the form of low stability, poor activity, high cytotoxicity, and hemolytic activity as well as high dosage for significant protection [52]. Biological molecules such as antibodies, proteins and DNA have flexibility and properties to make nanoparticles- biomolecule conjugates for application in biomedical science even AMPs have gained extensive abilities to combat plant and animal diseases [53]. For illustration, the peptide, VG16KRKP has not shown activity against intracellular *Salmonella typhi*, but after incorporation of the peptide specific residues with gold nanoparticle, the non-toxic Au- nanoparticles -conjugated VG16KRKP overcome low eukaryotic cell permeability of natural AMPs and efficiently internalize into cell, showing strong anti-*Salmonella* activity, through interaction of the peptide residues with LPS which then lyse the bacteria [54]. In another study, monoclonal antibody modified popcorn shaped gold nanoparticle selectively destroyed multidrug resistant *Salmonella typhimurium* DT104. The bacterial sample was exposed to 670 nm laser radiation for 25 min, and as a result almost 100% of *Salmonella* were killed in food samples due to high absorption of light and completely conversion to heat via the nonradiative properties resulting in photothermal lysis and caused irreparable bacterial cell damage [55]. Copper sulfide a cheaper alternative when conjugated with bovine serum albumin showed no killing activity in antibacterial tests for *Staphylococcus aureus* and *Escherichia coli.* However, after NIR exposure, the nanocomposite at low concentration exhibited strong NIR irradiation absorbance and can be used as an efficient photothermal conversion agent for bacteria ablation (80%) with a 980 nm laser within 10 min [56].

Tong et al. (2018) reported that the nanocomposite of DNA with Ag- nanoparticles and Graphene (ssDNA-AgNPs@GO) presented larger activity against Gram-positive and Gram-negative bacteria with low MIC compared to ssDNA-Ag-nanoparticles and GO alone [57]. The single strand DNA raised the cohesion strength between ssDNA-AgNPs@GO and bacterial cell membrane, temporarily, the even spreading of Ag- nanoparticles on graphene oxide is cooperative for connecting and covering bacteria too [57]. In another report, the oligonucleotides sequence Seq 3 coated on silver nanoclusters was shown to have bactericidal effects against bacteria in sub-micromolar range. This activity was mainly attributed to Seq 3 that have more cytosine which can bind with more silver than the other nucleobases but the activity is not only due to the amount of silver, but the DNA sequence, final structure and Ag arrangement are key players in the antibacterial effect [58]. The interaction of silver nanoparticles conjugated with egg-white protein with bacterial cells leading to reactive oxygen species creation, which prompt oxidative imbalance in the cell, result in the cell membrane deterioration and cell end, showing high antibacterial activity whereas the MIC for *E. coli* was 46 µg/mL while 6 µg/mL for *S. typhimurium* [59].

## 3. Nanoparticles as Drug Delivery Systems

One of the important uses of nanomedicine is drugs delivery to specific cells and receptors using nanomaterials [60]. Nanoparticles have potential to move through the blood stream, cross the biological barriers effectively and deliver drugs [61]. The transport of drugs to the site of infection is primarily based upon efficient loading of the drugs with the enhanced ability to penetrate cells and overcoming common barriers, as well as response triggered release of the drug [32]. Besides drug loading on nanoparticles, their release also needs to be controlled. A wide diversity of stimuli- reactive polymers have been used to advance nanosystems for drug delivery. These systems show drastic variations in response to various inducements because of creation or disturbing of secondary forces (electrostatic interactions, hydrophobic effects, hydrogen bonding, etc.), solubility, conformation, degradation, bond cleavage and reversibility [62].

There are two main modes for drug release in nano-drug delivery systems, locally chemical stimulated; or externally stimulated. The earlier can occur by simple diffusion and/or through diverse endocytic processes needing chemical and biochemical motivations (i.e., enzymatic activities, hydrolysis, pH, etc.). Otherwise, externally physical activated targeting is centered on external issues, for instance magnet, electrical, ultrasound, temperature and light [62,63]. For example, Gupta et al. (2004) showed that the surface coating of superparamagnetic iron oxide nanoparticles with hydrophilic-hydrophobic polymeric compounds (PEG, poloxamines, poloxamers) was significantly useful for relevant drug delivery by minimizing plasma protein adsorption to the nanoparticles and eliminating reticuloendothelial system uptake [64]. Food and Drug Administration accepted Visudyne, stimuli-responsive nanomedicine, which is consumed for photodynamic therapy [62,65].

As the initial illustration of pH triggered-receptive antibiotic secrete and to release in the specific site, the designation of the nanoparticles relies on acidity gradient of tissues such as skin, digestive tract, and environment inside cells [66]. Chitosan altered gold nanoparticles was connected to the liposomal surface to stop the undisciplined union of spherical vesicles. At low acidic PH rates, the attached gold nanoparticles separate from liposomes and the existence of microbial toxins damaged the liposome bilayer membranes to liberate antibiotics [67]. Ji et al. (2016) demonstrated the bacterial toxin triggered drug delivery [68]. Furthermore, in small molecule conjugated nanoparticles, the charges of both liposome and nanoparticles affect PH stimuli responsive connecting and disconnecting of nanoparticles. For example, liposome- and gold-nanoparticles bind with bacteria at bodily and acidic PH depending on charge distribution [67,69]. For enzyme responsive polymeric nanoparticles, Xiong et al. (2012) used lipase polymeric compound as a drug delivery carrier, once the layers of complex discerned the lipase liberating *S. aureus*, the layers are degraded to release vancomycin. Graphene-mesoporous silica surrounded by hyaluronic acid with overloaded ferromagnetic nanoparticles and ascorbic acid. When the conjugated compound accesses the infection location, bacteria release the hyaluronidase which in turn destroy hyaluronic acid. This disruption lead to ascorbic acid changing to hydroxyl radicals, followed by membrane devastation of bacteria [70].

## 4. Nanoparticles Cytotoxicity

**Figure 1:**
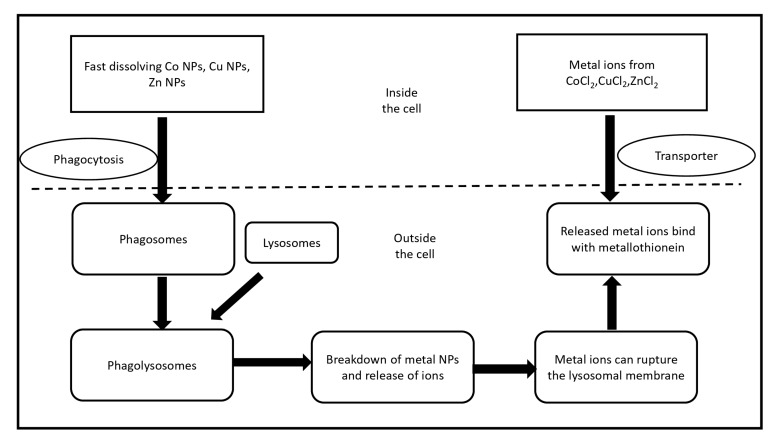
Schematic illustration for the different cytotoxic influence of rapid dissolving metal oxide nanoparticles and their specific metal chlorides. Nanoparticles cross the cell by phagocytosis forming phagosomes which fuse with spherical lysosomal organelle to arise phagolysosomes. In the low PH fluid of lysosome, analysis of nanoparticles and its metal ions can break lysosomal layer followed by the resulted free metal ions attach with metallothionein, while the membrane transporters are responsible for entering metal ions, then bind to metallothionein.

The toxicity of nanoparticles has remained a significant concern, which has limited their clinical applications. Creation of reactive oxygen species is one of chief mechanisms of nanotoxicity influenced by nanoparticle surface chemistry and surface charges [71]. There is no clear distinction among nanomaterials for their cytotoxicity, and there are several contradictory reports based on the design of experiments, types of nanomaterials, choice of cells etc. The capture of nanoparticles by endocytosis and enhancing intracellular death due to faint cell-nanoparticles adhesive interactions are considered acceptable explanation for increasing the cytotoxicity in case of uncoated nanoparticles. The high cytotoxicity in relation to increasing nanoparticles concentration, the surface chemical aspects of nanoparticles determine nanoparticle–cell interaction, adsorption way, and cell behaviour on contact [66]. Isabel et al. (2017) reported different types of metal nanoparticles against viability and morphology of cerebral cells [72]. However, it is important to note that these materials have been tested as drug vectors in other cell lines without affecting viability [73]. In a recent report, the effect of biogenic silver nanoparticles on breast cancer cell line (MCF-7) cells at specific IC50 doses resulted in apoptosis through increased ROS and decrease in membrane potency of mitochondria in addition to cell cycle arrest and DNA shattering causing oxidative burst and mitochondrial function failure in the way of MCF-7 death [74]. Jeong et al. (2018) showed the differential comparing in the cytotoxic effects and mechanism of toxicity of rapid dissolving metallic oxide nanoparticles (CuO, CoO, and ZnO) and their metal ions constituent using related epithelial cells for inhalation environment. The potential cytotoxicity of CoO NPs and CuO NPs showed similarities while as ZnO NPs showed a much less cytotoxicity, because of Zn-metallothionein that can behave as antioxidant, compared to their respective metal chloride [75]. This difference in toxicities may be caused by various intracellular absorption of these materials and their interactions as shown in Figure 1.

## 5. Potential Future Strategies against Bacterial Infections

There is a plethora of research regarding antibacterial efficacy of a wide variety of nanomaterials [76]. In spite of the appealing accomplishments of nanoparticles, the complete potential of nanotechnology in antimicrobial remedy is distant from getting drug administrative agencies approval [13]. However, until now no nanomaterials have been approved by Food and Drug Administration against systemic bacterial diseases as compared to other diseases such as cancer and this is mainly due to limited knowledge about the mechanism action of nanomaterials especially in humans [77].

In this regard, using the nanoparticles in inhibition of expression of target genes by nucleic acid-based molecules (microRNA, antisense oligonucleotide, and small interfering RNA) have shown effective increase in bacterial penetration and/or target delivery to infection sites [13]. Genomic analysis could be a valuable tool to study the whole or partial gene differentiation to give more insight about the associated mechanism of particular nanomaterials [20]. An important strategy to develop highly effective antibacterial agent can be based on the conjugation of multiple antibiotics in various mechanistic methods at one platform to interrupt the bacterial defense system and simultaneous delivery of more than one drug in the carrier against the same pathogen. Moreover, decrease of excessive excipients and medications ease drug-associated side influences and drug resistance [78]. For example, using multiple drugs in one composition for enhancing efficacy of the mixture by synergistic effects of combination (gentamicin and chloramphenicol) loaded on silver nanoparticles demonstrated better antibacterial effect compared to Ag- nanoparticles alone when tested against MDR *Enterococcus faecalis* associated with hospital-acquired infections [79,80]. In addition, the enhancing vascular permeability and retention at infection sites could be significant in the management of systemic nanoparticle drug delivery [13]. Another interesting avenue of research in drug discovery against bacterial infections could be repurposing of orphan drugs or drugs known to effect multiple targets in humans against MDR bacteria. For instance, the conjugation of auranofin, which is used for remedy of rheumatoid arthritis, with Poly(lactic-co-glycolic acid) (PLGA) nanoparticles displayed potent bactericidal effect against MDR *Streptococcus* species at 0.25 μM while the auranofin-nanoparticles was proficient of declining the bacterial cells around 4 times more than the free drug [81]. Biogenic nanoparticles, or nanomaterials coated with natural compounds of plants and animal origin has produced tremendous antibacterial effects which could be of potential future value. Moreover, newer classes of antibiotics, which have not yet been tested against bacteria in conjugation with nanomaterials could also result in increased efficacy and slow down the chances of development of resistance [82]. Premature drug delivery and use of nanostructured carriers functionalized with antigen-specific ligands exhibited improvement in the treatment of infectious diseases and in systemic delivery [78]. Recently, theranostics have gained significant importance in biomedical application and hold the promise of enabling pre-screening and therapeutics that may also be beneficial for antibacterial management [13]. However, the development of a practical and commercial application of nanotheranostic has not been yielded yet [82]. We anticipate that developing multiple functional modalities such as identification, isolation, and therapy at one platform will decrease the disease burden and will cause an overall improvement in the healthcare setting against bacterial infections.

## 6. Conclusions

In summary, here we emphasize multiple approaches that develop nanomaterials as therapeutic agents and drug cargo system. The adjustment of chemical functional group on the nanomaterials surface establish a wide range program for new antibacterial formation. The opportunity for fabrication of materials to realize great attraction between nanoparticles and bacteria, providing synergistic action with antibiotic, and decreases intensity of antibiotic resistance pathway. Finally, increasing the antibacterial efficacy, lowering of cytotoxicity, and avoiding immune cells recognition are basic requirement for fabrication a novel nanomaterial. The variety of nanomaterials presented in this review including antibiotics coated nanoparticles, small molecules conjugated nanoparticles, polymers and biomolecules stabilized nanoparticles, and nanocomposites have shown tremendous potential for biomedical applications and development of nanomedicine against bacterial infections.
